# Predicting Effects of Water Regime Changes on Waterbirds: Insights from Staging Swans

**DOI:** 10.1371/journal.pone.0147340

**Published:** 2016-02-10

**Authors:** Bart A. Nolet, Abel Gyimesi, Roderick R. D. van Krimpen, Willem F. de Boer, Richard A. Stillman

**Affiliations:** 1 Department of Animal Ecology, Netherlands Institute of Ecology (NIOO-KNAW), AA Wageningen, The Netherlands; 2 Resource Ecology Group, Wageningen University, Wageningen, The Netherlands; 3 Department of Life and Environmental Sciences, Faculty of Science and Technology, Bournemouth University, Talbot Campus, Poole, Dorset, United Kingdom; Estación Biológica de Doñana, CSIC, SPAIN

## Abstract

Predicting the environmental impact of a proposed development is notoriously difficult, especially when future conditions fall outside the current range of conditions. Individual-based approaches have been developed and applied to predict the impact of environmental changes on wintering and staging coastal bird populations. How many birds make use of staging sites is mostly determined by food availability and accessibility, which in the case of many waterbirds in turn is affected by water level. Many water systems are regulated and water levels are maintained at target levels, set by management authorities. We used an individual-based modelling framework (MORPH) to analyse how different target water levels affect the number of migratory Bewick’s swans *Cygnus columbianus bewickii* staging at a shallow freshwater lake (Lauwersmeer, the Netherlands) in autumn. As an emerging property of the model, we found strong non-linear responses of swan usage to changes in water level, with a sudden drop in peak numbers as well as bird-days with a 0.20 m rise above the current target water level. Such strong non-linear responses are probably common and should be taken into account in environmental impact assessments.

## Introduction

Human development is increasingly conflicting with nature [1, 2]. In order to prevent, reduce or offset negative effects on natural values, environmental impact assessments are often carried out in order to predict the environmental consequences of a new policy or development project. In ecology, however, these predictions are notoriously difficult, and usually are based on observed ecological relationships. However, such an approach is risky, especially when future environmental conditions fall outside the current range, because many ecological relationships are non-linear [3–5].

When considering effects on animal abundance, an alternative approach is to use models through which we can scale up from behavioural and ecological processes acting at individual level to patterns at population level. The reasoning behind this alternative approach is that these processes are shaped by evolution, and remain valid under new conditions. This approach has been successfully used to predict the impact of environmental developments (shell-fishing, building, sea level rise, and warm-water outflow from nuclear power stations) on numbers of migratory shorebirds wintering in estuaries [6–8], and to model foraging and departure behaviour of migratory birds at stopover sites [9, 10].

The number of migratory birds that make use of a wintering or staging site is often determined by the food supply [11]. However, the carrying capacity of a site is rarely a simple division of the amount of food present by the total consumption of a single individual, because many factors influence food availability and consumption rates, and these ecological relationships are often non-linear [12, 13]. In the case of waterbirds, for instance, water depth is an important determinant of available foraging grounds [14–16]. Hence, calculations of the carrying capacity of a site should consider the food availability dynamics and foraging costs, and not just the standing food biomass [17, 18]. Competition, both between and within species, and by direct and indirect processes such as behavioural interference and resource depletion, should also be taken into account [19–21]. Moreover, individuals may differ in important foraging attributes, such as reserve state, foraging efficiency or competitive ability [22]. More importantly, many of these processes interact, and these interactions are not revealed when studying them separately. Such processes acting at the individual level can be taken into account simultaneously by individual-based modelling (IBM), scaling up to patterns at the population level, such as animal distribution densities and carrying capacity [6]. Population traits arise from characteristics of the individuals and interactions among them, which can lead to a more realistic representation and even emerging properties of the study system [23].

In this study, we used an IBM to gain insight into the short-term effects of changes in water regime on the carrying capacity of a shallow lake for staging Bewick’s swans *Cygnus columbianus bewickii* during their autumn migration. Carrying capacity is here defined as the number of birds that can be supported by the available food supply during a stopover period. Water levels in the shallow lake are controlled and currently set at a target level. In order to simulate a more natural water regime, a more dynamic water level regime is envisaged, with lower target water levels in summer and higher target water levels in winter [24]. With the IBM we can evaluate the consequences of several water regime scenarios on the number of staging Bewick’s swans.

**Fig 1:**
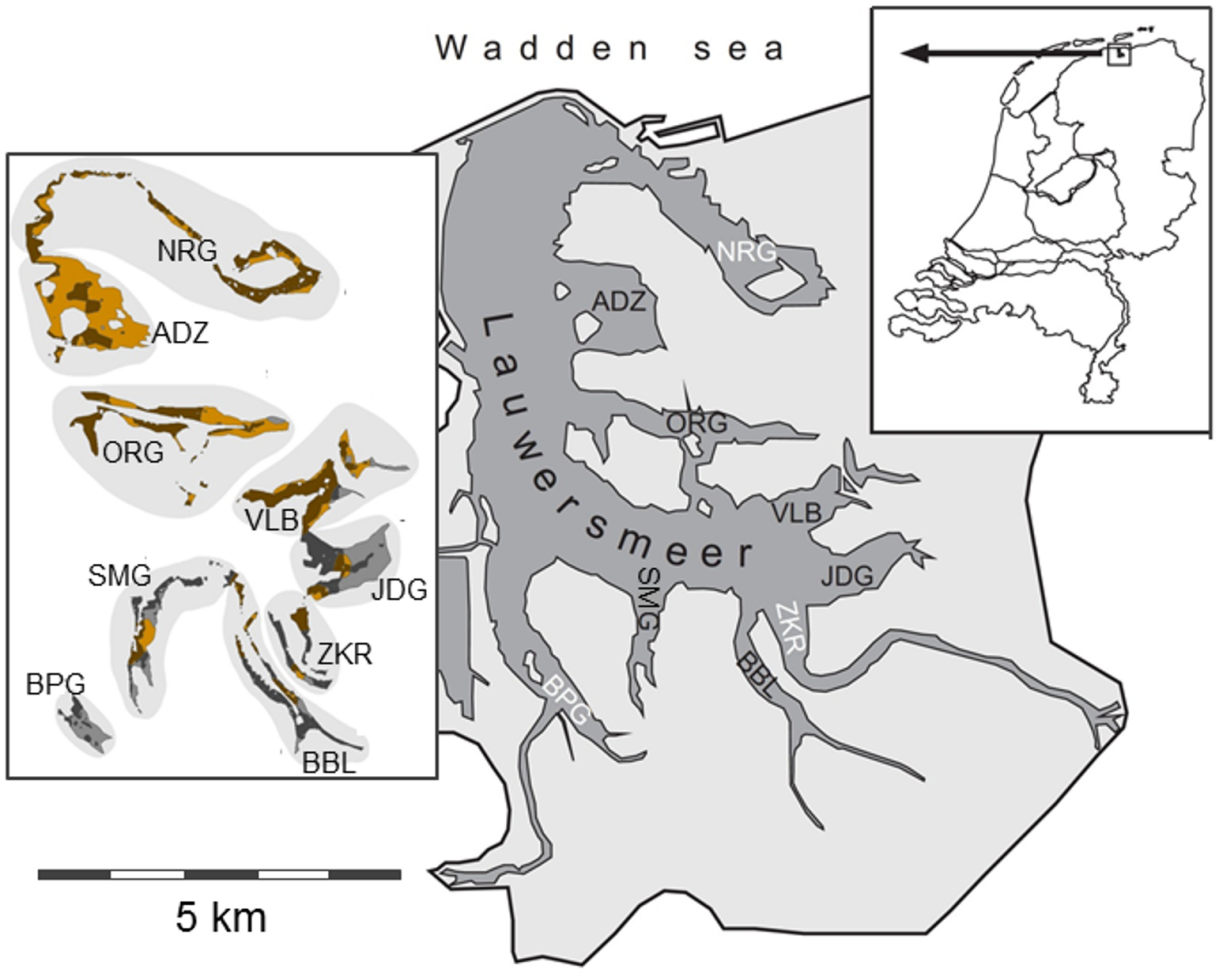
Map of the Lauwersmeer. Three letter abbreviations indicate the nine inlets. Inset to the right shows the location of the Lauwersmeer in the Netherlands. Inset to the left shows the patch types within inlets: sandy-shallow (light brown), sandy-deep (dark brown), clayey-shallow (light grey) and clayey-deep (dark grey). Parts of the lake that are shallower than 0.10 m or deeper than 0.86 m are not shown in this inset.

During migration, Bewick’s swans forage on aquatic macrophytes [25, 26]. Previous studies revealed that water depth determines resource accessibility and foraging costs [27], that intake rate and foraging costs are dependent on substrate as well as water depth [28], and that resource density and associated depletion determine changes in body condition of the swans [29]. Moreover, interference competition can negatively affect intake rates [30]. In those previous studies we were able to predict with hindsight (i.e. “retrodict”) swan numbers based on foraging processes in a single, well-studied inlet (BBL in Fig 1) [31–33]. In the current study, we scaled up to the whole lake, measuring resource density, water depth, and sediment type on patch type level across the entire lake. Next, we parameterized an IBM using published field and laboratory measurements (Table 1). With this model we inferred the distribution of the swans over the patches according to behavioural rules, assuming intake rate maximization under interference competition, and predicted staging swan numbers at the lake level. The model was calibrated by varying the number of immigrating individuals, and by fitting modelled cumulative swan numbers to observations. Using the calibrated model, predictions were made about what the effect of lower and higher target water levels would be on swan numbers staging in the lake in autumn. Rather than making predictions about specific future water regime scenarios, we aimed to deliver a proof of concept, i.e. demonstrate that this approach might be useful in an assessment of the impact of water regime changes on numbers of staging waterbirds.

**Table 1:** Parameter values used in the model, with symbols, units and references.

Parameter	Symbol	Unit	Value	Reference
**On pondweed beds (tuber foraging)**				
Water level pursued by water manager		m	-0.93**	a
Minimum water depth suitable for foraging		m	0.10	b
Maximum water depth for head-dipping		m	0.51	[32]
Maximum reachable depth		m	0.86	[27]
Tuber burial depth shape factor	*β*(Sand)	-	0.00077	[32]
	*β* (Clay)	-	0.00090	[32]
Energy density of food	*e*	J g^-1^	16866	[34]
Proportion of time spent foraging	*f*	-	0.66	[35]
Proportion of foraging time spent underwater	*φ*(Sand)	-	0.76	[32]
	*φ*(Clay)	-	0.72	[32]
Attack rate	*a*(Sand)	m^2^ s^-1^	0.00102	[34]
	*a*(Clay)	m^2^ s^-1^	0.000612	[32]
Handling time	*h*	s g^-1^	1.82	[34]
Benefit from area-restricted search	*b*	-	1.38	[36]
Assimilation efficiency	*q*	-	0.9	[34]
Metabolic rate while foraging	*c*_*f*_ (S-S*)	J s^-1^	45.1	[27]
	*c*_*f*_ (S-D*)	J s^-1^	56.7	[27]
	*c*_*f*_ (C-S*)	J s^-1^	58.6	[32]
	*c*_*f*_ (C-D*)	J s^-1^	73.7	[32]
Metabolic rate while resting	*c*_*r*_	J s^-1^	22.2	[34]
Metabolic rate while preening	*c*_*p*_	J s^-1^	33.8	[34]
Metabolic rate while flying		J s^-1^	204	[37]
Flight speed		m s^-1^	12.8	[34]
Mean flight distance (between inlets)		m	3317	c
Mean costs per flight (between inlets)		J	52699	c
Lean body mass		g	4660	[38]
Energy density of stores		J g^-1^	27.5 10^3^	[39]
Initial energy store of swans		J	14.3 10^6^	[40, 41]
Target energy store of swans		J	49.0 10^6^	[40, 41]
**On fields (sugar beet foraging)**				
Fraction of pondweed gain at switch to fields	*p*	-	0.65	[34]
Daily metabolizable energy intake	*MEI*_*b*_	J day^-1^	7.50 10^6^	[34]
Daily energy expenditure	*DEE*_*b*_	J day^-1^	2.61 10^6^	[34]

*S-S: sandy-shallow; S-D: sandy-deep; C-S: clayey-shallow; C-D: clayey-deep;

**above sea level (Normaal Amsterdam’s Peil, NAP).

References: a: Waterschap Noorderzijlvest; b: Personal observations; c: This study.

## Methods

### Study system

The study was carried out in National Park Lauwersmeer in the north of the Netherlands (53° 22’ N, 06° 13’E) in four autumns (2005–2008), under permits issued by Staatsbosbeheer. The Lauwersmeer is a former part of the Wadden Sea, which turned into a shallow freshwater lake (2000 ha) after dykes and sluices were built in 1969 [25]. The lake consists of nine inlets (former creeks), with a gradient from sandy to clayey sediments from north to south (Fig 1). Soon after the lake’s creation, fennel pondweed *Potamogeton pectinatus* appeared in the shallower parts (<1m) [42], and this remained the most common macrophyte species [43]. Fennel pondweed mainly overwinters by belowground tubers [44], and these energy-rich tubers are consumed by Bewick’s swans during autumn migration [25]. While foraging, the swans trample with their feet to loosen the tubers from the sediment, and subsequently head-dip or up-end to extract these tubers [45]. The foraging swans are accompanied by diving ducks, mainly pochards *Aythya ferina* and tufted ducks *Aythya fuligula*, that dive for tubers that are excavated by trampling of the swans [25]. Experiments showed that these diving ducks do not affect intake rates of the swans [46], and are therefore ignored. Transparency, measured with Secchi disc, was c. 0.5 m (data water board Waterschap Noorderzijlvest). After depletion of pondweed beds, which is also dependent on water levels, the swans switch to beet fields surrounding the National Park, or continue migration [32, 47].

### Field Data Collection

In October and November 2005–2007, and mid-October until mid-December 2008, swans were counted in the nine inlets from vantage points using a 20–60× telescope (Swarovski ATS 80 HD) on a near-daily basis (observation gaps, 6%, were linearly interpolated).

In early October of 2005–2008, sampling points were stratified among the nine inlets, and randomly located within inlets (10 points/inlet), with a minimum distance of 100 m between points to avoid autocorrelation [48]. At these points (1 m^2^), 12–16 sediment cores (0.10 m in diameter) were taken, sieving the upper 0.35 m of the sediment. For each sampling point, water depth (and current water level in the lake in order to calculate standardized water depth) and sediment type were recorded, and the dry mass of the tubers determined. Final tuber biomass density was measured by revisiting all sampling points after the swans had left the lake (November 2005–2007 and December 2008), and repeating the sampling procedure.

### Individual-Based Model

#### Framework

We used the MORPH IBM framework [49] to model the Lauwersmeer, where swans forage and move between pondweed patches in order to maximize fitness (see ‘forager variables’ for further details). The variables of the model framework are grouped into three categories: global variables, patch variables and forager variables (see below). Parameter values were based on previous studies (Table 1). The total number of bird-days was selected as the output variable, i.e. the sum of the daily number of swans that visited the lake. Model fit was evaluated by comparing predicted daily number of swans with the daily swan counts. For a description of MORPH according to the standard (“ODD”) protocol, see [49].

### Global variables

The model simulated two months (2005–2007: October—November; 2008: mid October—mid December) with a time step of one hour. Water level was recorded every 15 minutes by the water board (Waterschap Noorderzijlvest) in two different inlets (Zoutkamperril ZKR and Nieuwe Robbengat NRG, Fig 1) in the lake. We took hourly means of ZKR and NRG to calculate the average water level per hour. Water flows into the Lauwersmeer from the hinterland; above the target level of -0.93 m NAP (NAP being the reference level in the Netherlands, see footnote Table 1), water is let out by a sluice into the Wadden Sea at low tide.

### Patch variables

Patches (of pondweed) were characterized by combinations of two sediment classes and two water depth classes, resulting in four different patch types (Fig 1). Sediment *s* was classified into sandy and clayey [17]. Water depth, standardized to the water level targeted by the water board (Waterschap Noorderzijlvest), was divided into shallow (0.10 m-0.51 m) and deep (0.52 m-0.86 m), based on the feeding behaviour of the swans (head-dipping *vs* up-ending). Standardized water depths shallower than 0.10 m and deeper than 0.86 m were basically too shallow and too deep, respectively, for fennel pondweed to grow (A. Gyimesi & B.A. Nolet, unpubl. data), therefore, areas out of the range 0.10 m– 0.86 m were excluded from the model. The average water depths were respectively 0.33 and 0.62 m for the shallow and deep water classes. Because water levels fluctuated, the actual water depth (*d* in m) per patch varied. Actual water depth was calculated by correcting for the observed hourly water level (described under Global variables), and accessibility and foraging costs were adapted accordingly (see below). The size of each patch type in each inlet was calculated using ArcGIS (version 9.2, ESRI, Redlands, USA). The total number of patches was 30 since not all patch types were present in all inlets (Fig 1).

In order to calculate initial tuber biomass density *D* (g m^-2^) per patch type, the mean tuber biomass of all cores per patch type, irrespective of inlet, was taken (Table 2). This variable was year-specific, because of differences in tuber biomass among years [50]. No change in tuber density besides depletion was modelled; in autumn the tubers stop growing as aboveground plant parts are already washed away before the visit of the swans, and tuber mortality is negligible over the short exploitation period [51].

**Table 2:** Tuber densities before and after the swan autumn stopover period. Measured tuber densities (mean ± SE, (N)) in four patch types in the Lauwersmeer in four years. Samples were taken in autumn shortly before the arrival of swans (“Initial”) and after swan departure (“Final”). N is number of sampling points.

Year	2005		2006		2007		2008	
Patch Type	Initial	Final	Initial	Final	Initial	Final	Initial	Final
Sandy—Shallow	13.2 ± 2.2 (43)	8.8 ± 1.4 (43)	19.4 ± 2.2 (39)	11.4 ± 1.2 (39)	13.3 ± 2.0 (28)	8.5 ± 0.7 (28)	14.7 ± 2.1 (28)	11.6 ± 1.4 (28)
Sandy—Deep	16.4 ± 3.5 (5)	20.6 ± 1.6 (5)	13.9 ± 1.6 (6)	7.5 ± 1.5 (6)	7.9 ± 1.9 (12)	7.0 ± 1.6 (12)	17.5 ± 4.9 (11)	15.4 ± 3.1 (11)
Clayey—Shallow	9.9 ± 2.6 (31)	9.2 ± 1.4 (31)	14.2 ± 2.2 (35)	14.2 ± 2.3 (35)	14.9 ± 2.0 (30)	10.0 ± 1.6 (30)	19.3 ± 3.5 (33)	14.8 ± 2.6 (33)
Clayey–Deep	22.2 ± 4.9 (11)	18.1 ± 5.9 (11)	27.0 ± 4.9 (10)	24.4 ± 4.1 (10)	12.4 ± 1.4 (15)	12.5 ± 1.7 (15)	30.7 ± 6.0 (13)	22.6 ± 4.0 (13)
Weighted Mean	14.5	13.2	17.7	12.3	12.0	8.8	18.2	14.5

The proportion of accessible tuber biomass *A*(*d*) was calculated per patch depending on actual water depth *d*. If, at a given time step, a patch was shallower than 0.51 m or deeper than 0.86 m, *A*(*d*) = 1 or 0, respectively [27]. For all depths in between, accessibility was calculated as [derived from 32]:

$$A(d)=\beta (s).{\left((0.86-d)\times 100\right)}^{2}$$ (1)

where *β*(*s*) is a tuber burial depth shape factor depending on sediment type (Table 1).

The attack rate *a*(*s*) (m^2^ s^-1^), the proportion of foraging time spent with their heads underwater *φ*(*s*), and the metabolic rate while foraging *c*_*f*_(*s*) (J s^-1^) differed among the four patch types. The attack rate and proportion of foraging time spent underwater depended on sediment type, because it takes the swans more time to extract tubers from clayey soils compared to sandy soils [28, 32]. The metabolic rate while foraging depended on both sediment type and actual water depth, with foraging on clay and in deep water being more costly [27, 28].

### Forager variables

The model contained a total of 3000–6000 swans (corresponding to the range in numbers present in the northern Netherlands in autumn 2003–2007; [52]), comprised of super-individuals of 20 swans each. Bewick’s swans rarely operate in flocks smaller than this, and usage of super-individuals speeds up the simulations considerably [53]. At the start of a simulation no swans were present in the model. Arrival date varied among years in the model, reflecting differences in derived arrival dates among years (2008 being particularly late; Table 3). This is relevant because water level also varied with date. The influx was modelled to consist of two peaks (simulating non-breeders and breeders; [25]), the second peak (half the size of the first one) occurring 14 days after the first peak. (Table 3)

**Table 3:** Immigration peaks of swans to the model. Immigration dates of the swans followed a normal distribution with a mean and standard deviation (= 3 days) based on arrival dates derived from the observed numbers in the field. Arrivals (and departures) in the field were approximated by taking the difference in daily bird numbers counted at subsequent days (Fig 2).

Year	2005	2006	2007	2008
First peak	19 Oct	18 Oct	19 Oct	9 Nov
Second peak	2 Nov	1 Nov	2 Nov	23 Nov

Tuber intake rate *I* (g s^-1^) depended on tuber biomass density, sediment type, actual water depth, and swan density (see below), and was modelled as a type II functional response [34, 54]. By moving adaptively within patches, swans make use of the positive spatial autocorrelation in the abundance of pondweed biomass, encountering high-density patches more often, and low density patches less often than their frequencies on offer. This results in a higher long-term intake rate (benefit from area-restricted search *b* = 1.38) [36]. Bewick’s swans refuelling on tubers of pondweed are time-limited, and the model swans could therefore maximally forage an estimated 66% of the time (proportion of time spent foraging *f* = 0.66) [35]. We included interference competition in the model by reducing the intake rate with higher densities of super-individuals in a certain patch according to the results of Gyimesi *et al*. [30], who modelled the effect of interference competition on tuber intake rates of Bewick’s swans at the Lauwersmeer using a polynomial equation for the relative intake rate *i* as function of swan density *N* (m^2^):

$$i\left(N\right)=-530344\times N^{4}+60100\times N^{3}-1976.3\times N^{2}-0.7284\times N+1$$ (2)

Tuber intake rate was thus modelled as:

$$I=f.b.i\left(N\right).\varphi \left(s\right).\frac{a\left(s\right).A\left(d\right).D}{1+a\left(s\right).h.A\left(d\right).D}$$ (3)

where *h* is the handling time (s g^-1^).

The metabolizable energy intake rate is the tuber intake rate multiplied by the energy density of tubers *e* (J g^-1^) and metabolizability *q* [34]. The energy costs while on the pondweed beds were calculated as the proportion of time spent foraging (*f*) times the costs of foraging (*c*_*f*_ in J s^-1^), and the proportion of time not spent foraging (1 –*f*) times the costs of non-foraging, taken as the average of the costs of resting (*c*_*r*_ in J s-^1^) and the costs of preening (*c*_*p*_), the main alternative activities [35]. As energetic costs were determined under field conditions [34], they were assumed to include heat increment of feeding, and no further correction was made. Therefore, the (net) energy gain rate at tubers *G*_*t*_ (J s^-1^) was:

$$G_{t}=q.e.I-\left(f.c_{f}+\left(1-f\right).\frac{c_{r}+c_{p}}{2}\right)$$ (4)

At each time step, each super-individual could make the decision to stay at the same patch, go to another patch, or to emigrate. These decisions were based on the expected obtainable fitness (i.e., gain rate or net energy intake rate) for each patch, assuming perfect knowledge of the swans. Because the model uses time steps of one hour, the costs and time of travelling between patches were simplified. Travelling time between patches was dependent on a threshold distance. Moving to patches that were <3316 m (the mean distance between inlets) from the current patch did not take any time nor energy, whereas travelling by flying to patches >3316 m from the current patch took one time step (i.e. 1 h, including settling time) and cost 3316/12.8×0.204 = 53 kJ (see Table 1 for details).

The initial energy store (J) of arriving swans was based on their abdominal profile index (*API*). We assumed a lean body mass of 4660 g [38], and that stores had an energy density of 27.5 10^3^ J g^-1^ [39]. At arrival, *API* = 2 [41], which was converted into individual swan mass (5182 g) using an equation based on body mass and tarsus length [40, 55]. The swans left the model either when they reached their target energy store (*API* = 5 or 6441 g) [41], or when the expected fitness of emigration (foraging outside of the model area, i.e. on beet fields) was above the fitness that could be obtained from foraging on pondweed patches. The swans tended to switch to beet fields when the gain rate of tuber-feeding *G*_*t*_ had fallen well below the gain rate they could attain at beet fields *G*_*b*_ (i.e., *G*_*t*_ ≤ *p*. *G*_*b*_; *p* = 0.65 [34]), as an effect of the perceived higher predation risk at beet fields. Therefore, emigration occurred when the gain rate of tuber-feeding, *G*_*t*_ dropped to:

$$G_{t}<\left(p.\left(MEI_{b}-DEE_{b}\right)\right)/86400$$ (5)

where *MEI*_*b*_ is the daily metabolizable energy intake rate on beet fields (J day^-1^), and *DEE*_*b*_ is the daily energy expenditure on beet fields (J day^-1^) [34].

### Simulations

As stated above, water level, tuber density and time of arrival varied with year in accordance with observations. For each year, we further varied, in steps of 500, the total number of incoming swans to calibrate the predicted staging swan numbers against the observations, in terms of pattern of swan presence, peak numbers and total bird-days.

A sensitivity analysis (see S1 File) showed that the model outcome in terms of total number of bird-days was robust to changes in parameter values except for changes in water depth (Figure C in S1 File). Hence, we subsequently simulated the effect of changes in water regime (i.e., target water level) on the total number of bird-days. Water is drawn from the Lauwersmeer whenever its water levels are higher than the target water level. However, in practice this is only possible when the water level in the Wadden Sea is lower than that in the Lauwersmeer. This usually occurs during low tide, except in periods of neap tide when the water level in the Wadden Sea tends to remain higher than in the Lauwersmeer even during low tide. This means that a given target water level cannot always be maintained. Whenever water level in the Wadden Sea was higher than the water level in the Lauwersmeer, the latter gradually rose, because drawdown from the Lauwersmeer was not possible. When the water level in the Wadden Sea permitted drawdown, and the water level in the Lauwersmeer was above the target level, the water level was lowered until the target water level was reached (Figure A in S1 File). Importantly, the dynamics of water levels above target water levels depend on the chosen target water level. This is because drawdown might already be possible with a higher target water level before being possible with a lower target water level, or continue with a lower target water level after being stopped with a higher target water level. In order to account for this effect, we modelled water levels in the Lauwersmeer in response to observed water levels in the Wadden Sea (at the sluice, provided by the water board, Waterschap Noorderzijlvest). The best least-squares fit was obtained with a rate of decrease in water level at drawdown of 0.15 m h^-1^ and a rate of increase during water build-up of 0.01 m h^-1^. These rates explained a good proportion of the observed variation in water levels (Figure B in S1 File; R^2^ = 0.63, 0.63, 0.64, 0.70, for 2005–2008, respectively). We varied target water levels between -0.73 m and -1.13 m NAP in steps of 0.10 m, using these modelled water levels.

## Results

**Fig 2:**
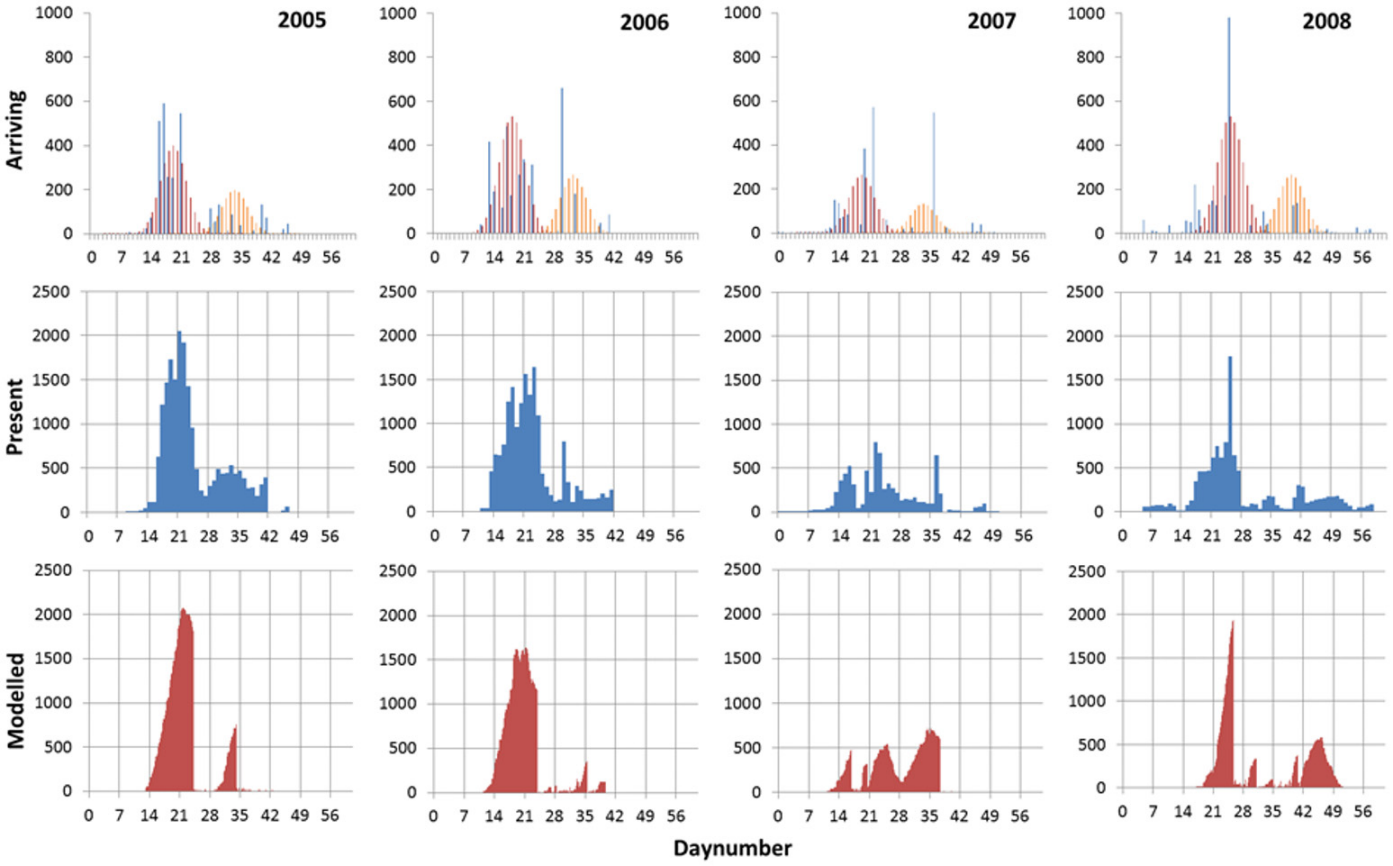
Autumn stopover of Bewick’s swans in the Lauwersmeer in four years. Top row gives the number of arriving swans, derived from the day-to-day difference in counted numbers of swans (blue, only positive values given), and modelled in two peaks (red and orange). Middle row gives the numbers of swans counted each day on the lake. Bottom row gives one example run of the daily number of swans modelled with a target water level of -0.93 m above sea level (NAP, see footnote Table 1).

The best fit between observed and modelled swan numbers was obtained with incoming swan numbers varying among years, from a minimum of 3000 in 2007, a maximum of 6000 in 2006, and an intermediate number of 4500 in both 2005 and 2008. Those incoming numbers resulted in a good agreement between observations and model outcome in the pattern of swan presence. In 2005, 2006 and 2008, the daily pattern was characterized by a large peak in swan presence followed by a smaller peak about 2 weeks later, whereas in 2007 three smaller peaks 1–2 weeks apart were observed. These general patterns were captured by the model (Fig 2).

For 2005 and 2006, the model predicted that the (super-)individuals arriving in the first peak stayed on average longer than those of the second peak (2005: 4.22 ± 0.17 and 2.52 ± 0.55 days; 2006: 2.95 ± 0.10 and 0.84 ± 0.10 days; mean ± SD, N = 4), whereas for 2007 and 2008 it was the other way around (2007: 2.75 ± 0.06 and 4.11 ± 0.17 days; 2008: 2.16 ± 0.05 and 3.14 ± 0.15 days). Accordingly, on average, (super-)individuals reached higher energy stores (expressed as percentage of the target energy store) in the first peak in 2005 and 2006 (2005: 69 ± 3 and 16 ± 5%; 2006: 89 ± 2 and 8 ± 3%), whereas in 2007 and 2008 there was not much difference between the (super-)individuals of the first and second peak (2007: 55 ± 14 and 68 ± 5%; 2008: 44 ± 11 and 42 ± 15%).

**Fig 3:**
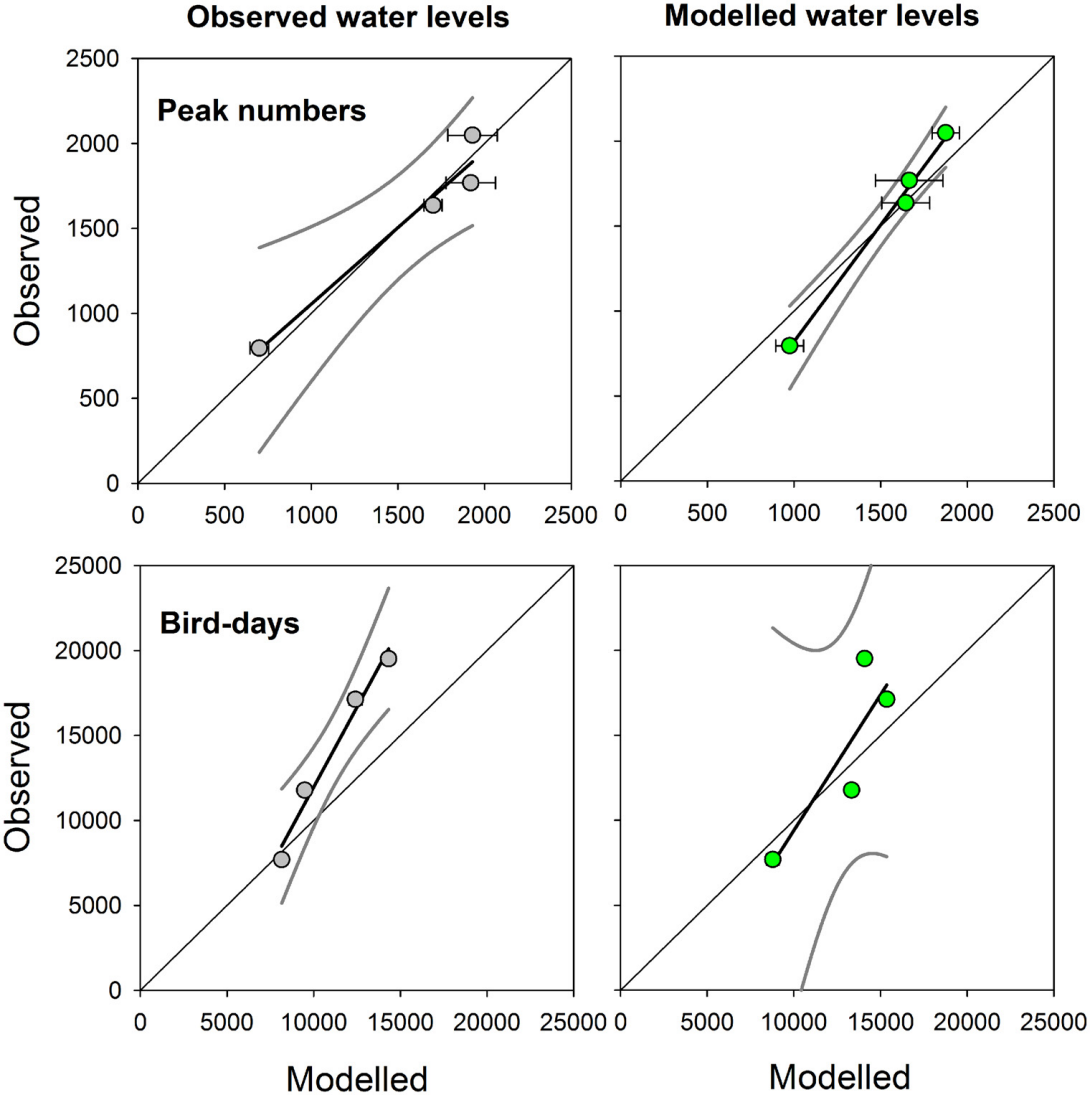
Comparison of observed and modelled Bewick’s swans numbers during autumn stopover in the Lauwersmeer in four years. Swan numbers were either expressed in maximum daily numbers (“peak numbers”) or in the sum of daily numbers (“bird-days”). Water levels were either observed or modelled with the current target water level (-0.93 m NAP, see footnote Table 1). Lines are *y* = *x* and linear regressions with 95%-confidence intervals. Error bars indicate SD (*N* = 4).

Also, in terms of aggregate numbers such as peak numbers and bird-days, the agreement between counts and model outcomes was satisfactory. Indeed, no significant differences between observed and modelled peak numbers nor between observed and modelled bird-days were apparent (paired t-test, Table 4). Also, for peak numbers, the intercept of the linear regression was not significantly different from 0 and the slope not significantly different from 1 (Table 4) when plotting the observed numbers against the numbers modelled under the observed water levels (Fig 3, left panels). It should be noted however that sample size (numbers of years) is limited, so the power to detect such a difference is low. When using water levels modelled under the current target water level of -0.93 m NAP, for peak numbers, the intercept was significantly different from 0 and the slope from 1 (Table 4), but even in this case, over the range of observed peak numbers, there was a good agreement with the modelled peak numbers (Fig 3 upper right panel). For bird-days, the slope was however significantly larger than 1 when plotting the observed numbers against the numbers modelled under the observed water levels (Table 4).

**Table 4:** Comparison of observed and modelled Bewick’s swan numbers during autumn stopover in the Lauwersmeer in four years. Results of paired *t*-tests and linear regressions of observed against modelled numbers, for peak numbers and bird-days separately. Significant results in bold.

	Paired *t*-test			Linear regression					
	*t*_3_	*P*	*R*^2^	Intercept	LL	UL	Slope	LL	UL
Peak numbers									
Obs vs. Mod(Obs)	-0.006	0.99	0.95	155	-577	888	0.90	0.45	1.35
Obs vs. Mod(Mod)	0.30	0.79	0.99	**-554**	**-988**	**-120**	**1.38**	**1.10**	**1.65**
Mod(Obs) vs. Mod(Mod)	0.21	0.85	0.95	-689	-1825	448	1.46	0.74	2.18
Bird-days									
Obs vs. Mod(Obs)	2.25	0.11	0.98	-6883	-34680	21564	**1.88**	**1.22**	**2.53**
Obs vs. Mod(Mod)	0.70	0.53	0.74	-6558	-14327	561	1.60	-0.54	3.74
Mod(Obs) vs. Mod(Mod)	-1.88	0.16	0.59	1410	-16939	19760	0.75	-0.65	2.15

Obs: observed; Mod(Obs): modelled with observed water levels; Mod(Mod): modelled with modelled water levels at current target water level of -0.93 m below sea level (NAP, see footnote Table 1).

LL: lower limit, UL: upper limit of 95%-confidence interval.

**Fig 4:**
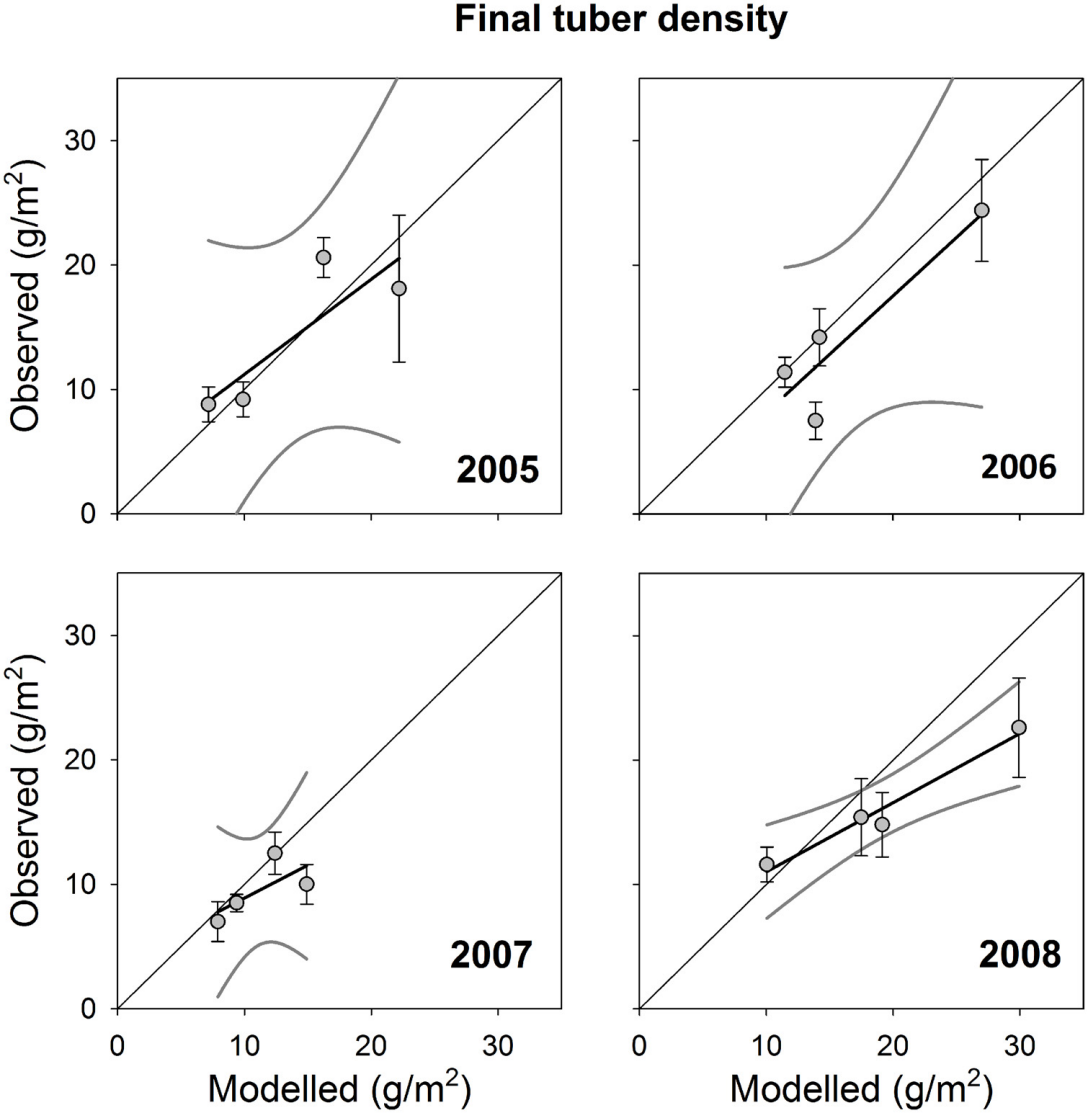
Comparison of observed and modelled tuber biomass density after swan stopover in autumn in the Lauwersmeer in four years in four patch types. Patch types were characterized by sediment type (sandy or clayey) and standardized water depth (shallow or deep). Lines are *y* = *x* and linear regression with 95%-confidence intervals. Error bars indicate SE for observations and SD (*N* = 4) for model predictions (the latter hidden by symbols). Modelled with observed water levels.

Another way to test the performance of the model is to compare observed and modelled tuber biomass densities after the swans have left the lake. Again, the agreement between measurements and model outcomes was generally satisfactory, with no significant differences between observed and modelled final tuber biomass densities (paired t-test, Table 5). Apart from 2008, the intercept of the linear regression was not significantly different from 0 and the slope not significantly different from 1 (Table 5) when plotting the observed final tuber density against the ones modelled under the observed water levels (Fig 4). Again, it should be noted that sample size (numbers of classes) is limited, so the power to detect such a difference is low.

**Table 5:** Comparison of observed and modelled final tuber biomass density after autumn swan stopover in the Lauwersmeer in four years. Results of paired *t*-tests and linear regressions of observed against modelled (with observed water levels) final tuber biomass densities. Significant results in bold.

	Paired *t*-test			Linear regression					
	*t*_3_	*P*	*R*^2^	Intercept	LL	UL	Slope	LL	UL
2005	0.16	0.88	0.72	3.56	-12.65	19.76	0.76	-0.31	1.84
2006	-1.51	0.23	0.83	-1.25	-18.18	15.68	0.94	-0.02	1.89
2007	-1.48	0.23	0.50	3.60	-10.12	17.32	0.53	-0.67	1.73
2008	-1.63	0.20	0.96	**5.44**	**0.53**	**10.37**	**0.56**	**0.32**	**0.80**

LL: lower limit, UL: upper limit of 95%-confidence interval.

**Fig 5:**
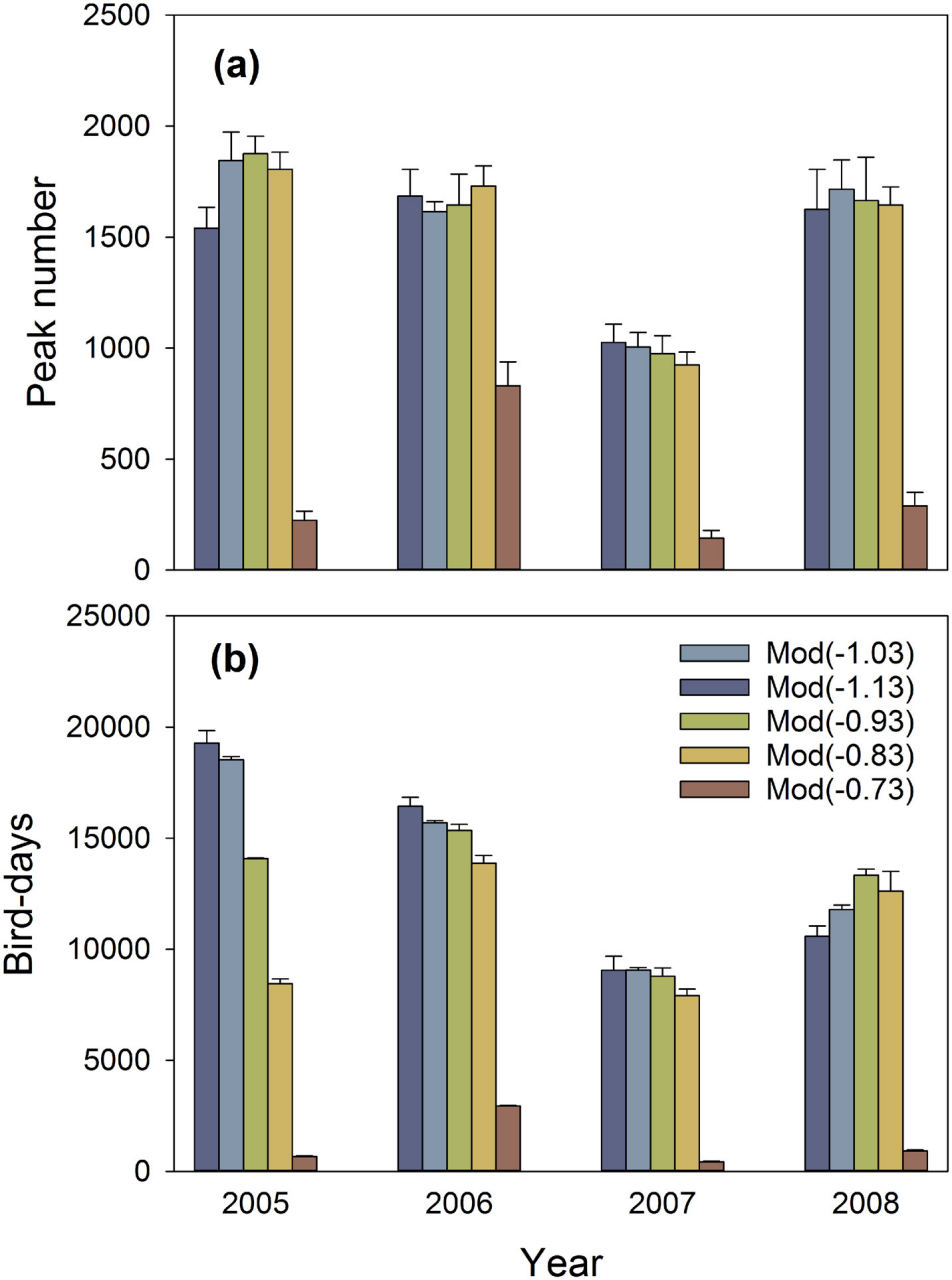
Modelled numbers of staging Bewick’s swans at varying target water levels in four years. Swan numbers were either expressed in maximum daily numbers (“peak numbers”) or in the sum of daily numbers (“bird-days”). Water levels were modelled with a target water level varying between -0.73 m and -1.13 m above sea level (NAP, see footnote Table 1). Error bars indicate SD (*N* = 4).

The water regime was changed by altering the target water level. Target water levels 0.10 m or 0.20 m lower (down to -1.13 m NAP) had little effect on peak numbers and bird-days; this can be seen in Fig 5 by comparing the predicted peak numbers and bird-days with those predicted under the current target water level (green bars). A slightly higher (+0.10 m) target water level (-0.83 m NAP) did not have much of an effect either. However, raising the target water level even more (+0.20 m) to -0.73 m NAP led to a dramatic decrease in the numbers of swans staging at the lake, both measured in peak numbers (Fig 5a) and in bird-days (Fig 5b).

## Discussion

Previous research demonstrated, in qualitative terms, that more Bewick’s swans were, in total, staging in inlets where they could obtain the highest net energy intake rate [17] and that yearly variation in staging swans in the lake was to a large extent explained by annual differences in tuber biomass density and water level fluctuations [33]. Also in quantitative terms, previous research was successful in predicting (or more precisely “retrodicting”) how many swan-days were spent in a well-studied inlet, taking temporal and small-scale spatial differences in tuber biomass density into account [25, 31, 32]. The current study extends this to a prediction of swan usage of the whole lake, depending on the tuber biomass on offer, the water level fluctuations, and the timing and numbers of arriving swans. We modelled the swan usage as occurring in two peaks: while the first peak was accurately predicted, the second peak tended to be underestimated. Overall differences were however not significant, therefore the study demonstrates that—based on annual differences in both tuber biomass density and water level fluctuations—we can “predict” the total number of swans as well as the temporal pattern of swan usage of the lake, as a whole.

Individual-based models are parameter-rich [56, 57]. This type of modelling is however suitable when resource supply and availability are temporally and spatially varying. These dynamics, further enhanced by resource depletion, can cause different individuals to experience different fuelling trajectories. In our case, for example, the timing of arrival determined resource levels and water levels encountered, and hence affected whether swans left the lake because they had completely refuelled or because their net energy intake rate fell below the critical threshold level. Arrival time of the swans is determined by conditions further upstream the migratory trajectory [58], and they are therefore largely unable to tune their arrival to favourable water levels. Overall residence time (days; mean ± SD, N = 4) of swans in the model (2005: 3.83 ± 0.06; 2006: 2.60 ± 0.12; 2007: 3.27 ± 0.05; 2008: 2.43 ± 0.07) is in line with observed minimum residence times derived from marked individuals (1995: 1.82 ± 1.56, N = 115; 1996: 2.24 ± 2.31 days, N = 74) (B.A. Nolet, unpublished data). However, individual differences in departure conditions were reflected in the different staging durations and final energy stores predicted for swans arriving in the first or second peak, and in different years.

Individual-based models are also known for their emerging properties [23]. In fact, the pattern of stopover use and the bird-days spent at the lake are emerging properties. We had to assume a large annual variation in the number of arriving swans to replicate the yearly patterns. Such variation is also apparent in autumn counts on migration sites of passing Bewick’s swans (see www.trektellen.nl; hourly mean observed number passing in autumn against modelled number of arriving swans: *R* = 0.878, one-tailed *P* < 0.07, *N* = 4), suggesting that this interannual variation is realistic.

Swans were assumed to have complete information about their fitness in all patches in the system, but because tubers of fennel pondweed are not visible for the swans, assuming incomplete information might be more realistic. However, red knots (*Calidris canutus*), that also feed on hidden prey items, behave as if they have complete knowledge on food distribution [59]. On the other hand, a modelling study of white-fronted Geese (*Anser albifrons*) foraging on crop remains found that a model assuming incompletely informed foragers resulted in the best fit with observed data [60]. So, the amount of information Bewick’s swans actually have on the food distribution and how this influences their decision making requires further investigation.

Having developed the individual-based model, we can actually predict the dynamics of swan stopover. It should be noted that we only modelled short-term changes in target water levels. In the longer term, we expect the submerged plants like fennel pondweed to respond to new target levels also, which will affect the surface area and biomass density of patches, and hence the stopover dynamics of the swans.

Our study suggests strong non-linear responses at the population level to changes in water regime and that, beyond a certain threshold, small changes in water level will have a large impact on the numbers of staging waterbirds. Part of this non-linearity may be due to our modelling of shallow and deep patches. However, the largest drop in staging swan numbers occurred when the water level rose to such height that even the shallow patches became nearly inaccessible to the swans. This finding can be important for the quantitative evaluation of management options at our study wetland. Further research using this or another framework to develop individual-based models might reveal this non-linearity to be an important aspect in other wetlands also.

## Supporting Information

S1 File Water level in Lauwersmeer and Wadden Sea in autumn in four years (Figure A in S1 File), modelled against observed hourly water levels in the Lauwersmeer in autumn in four years (Figure B in S1 File), sensitivity analysis (Text in S1 File) and elasticity of 19 parameters for the total number of bird-days (Figure C in S1 File). (DOCX)
