# Supplementary material for: Predicting Effects of Water Regime Changes on Waterbirds: Insights from Staging Swans
Source: PLoS One. 2016 Feb 10;11(2):e0147340. doi: 10.1371/journal.pone.0147340 (PMC4749328; doi:10.1371/journal.pone.0147340)
Supplement: S1 File — (DOCX) [file pone.0147340.s001.docx]

**Supporting Information**

**Predicting Effects of Water Regime Changes on Waterbirds: Insights from Staging Swans**

Bart A. Nolet^1,#a*^, Abel Gyimesi^1,#b^, Roderick R. D. van Krimpen^1,2^,

Willem F. de Boer^2^, Richard A. Stillman^3^

^1^Department of Animal Ecology, Netherlands Institute of Ecology (NIOO-KNAW), P.O. Box 50, 6700 AA Wageningen, The Netherlands

^2^Resource Ecology Group, Wageningen University, Wageningen, The Netherlands

^3^Department of Life and Environmental Sciences, Faculty of Science and Technology, Bournemouth University, Talbot Campus, Poole, Dorset, BH12 5BB, UK

^#a^Currently also at: Computational Geo-Ecology, Department of Science, Institute for Biodiversity and Ecosystem Dynamics, University of Amsterdam, P.O. Box 94248, 1090 GE Amsterdam, The Netherlands

^#b^Current address: Department of Bird Ecology, Bureau Waardenburg, Culemborg, The Netherlands

**
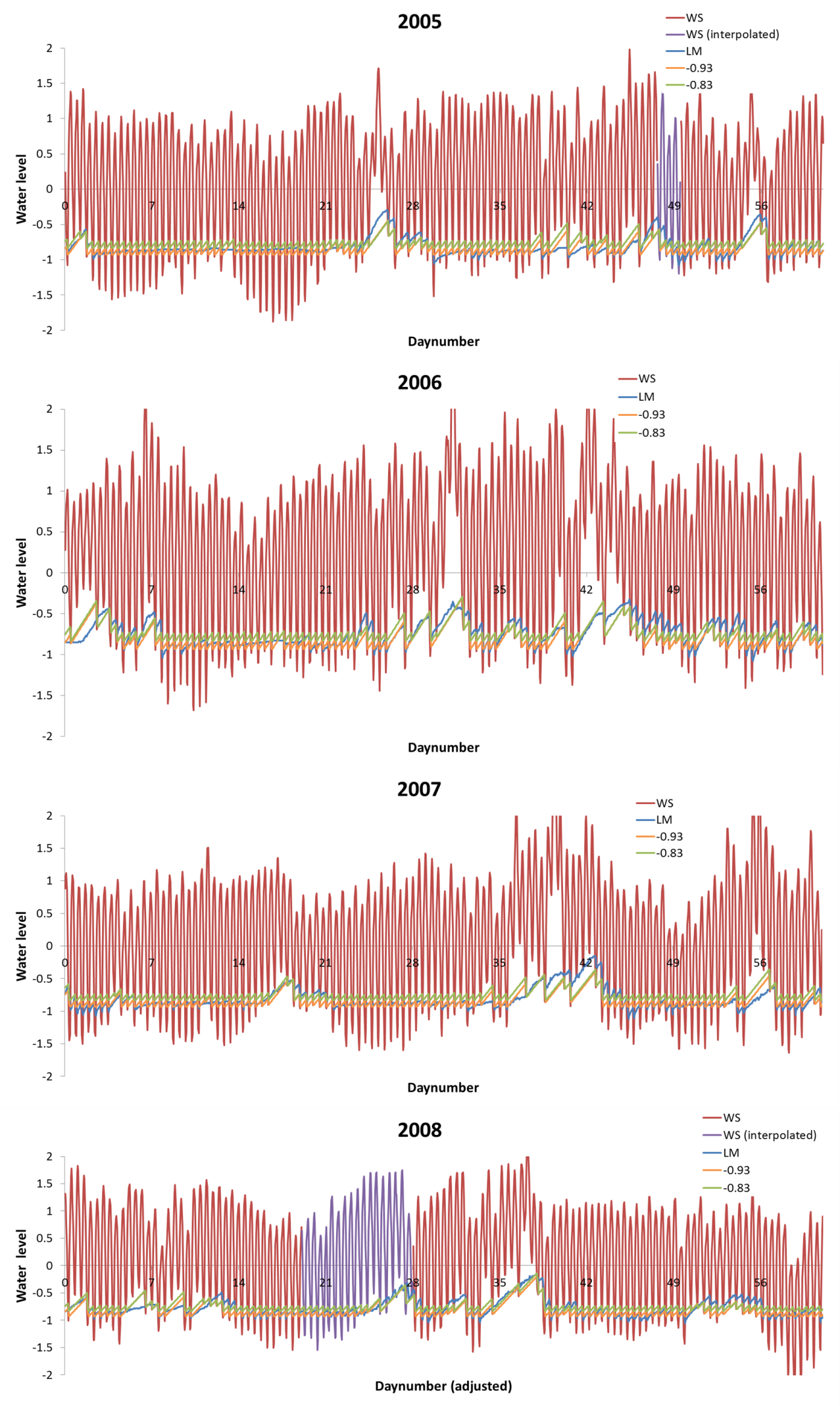
**

**S1 Fig. A**(at previous page) **Water level in Lauwersmeer (LM, blue) and Wadden Sea (WS, red, or purple when interpolated) over two month period in autumn in four years.** Water is drawn down from the Lauwersmeer whenever the water level is higher than the target level (-0.93 m) and that in the Wadden Sea (where it flows into). Water levels were modelled at target levels of -0.93 m (orange) and -0.83 m (green) with an increase rate of 0.01 m h^‑1^ and a decrease rate of 0.15 m h^‑1^. All water levels expressed in m relative to NAP (see footnote Table 1).

**
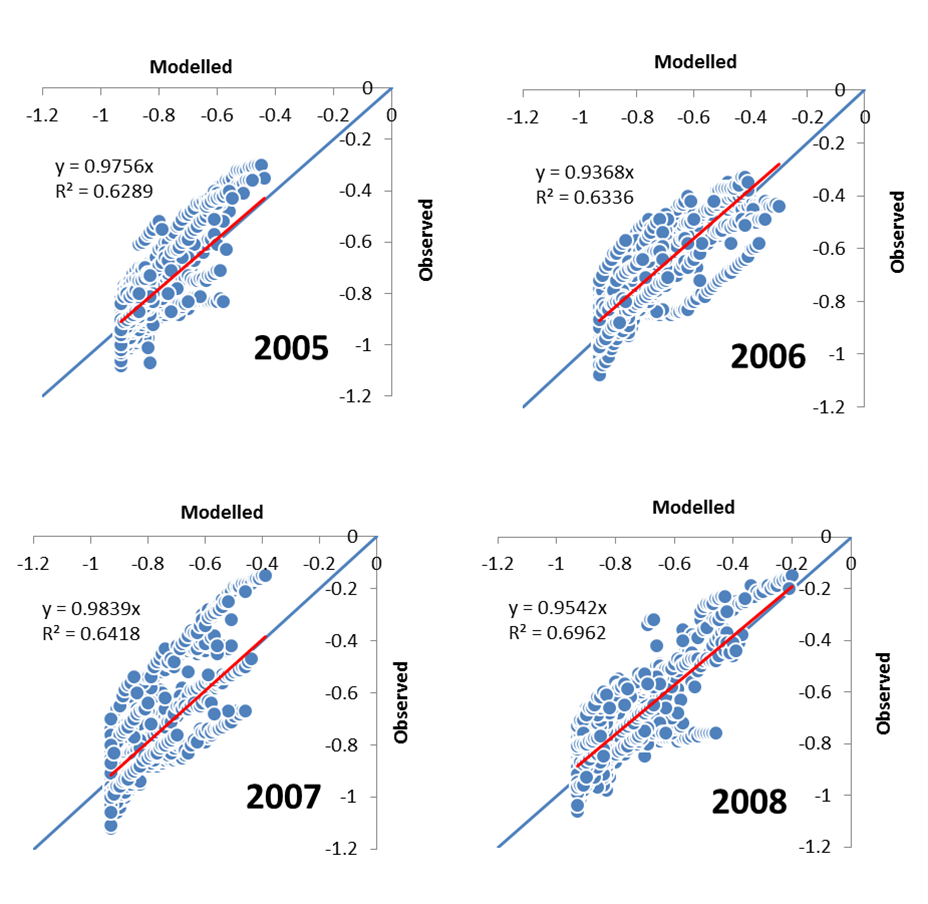
**

**S1 Fig. B Modelled against observed hourly water levels in the Lauwersmeer in two month periods in four years.** Water is drawn down from the Lauwersmeer whenever the water level is higher than the target level (-0.93 m) and that in the Wadden Sea (where it flows into). Hourly water levels were modelled with an increase rate of 0.01 m h^-1^ and a decrease rate of 0.15 m h^-1^. All water levels expressed relative to NAP (see footnote Table 1). Line *y* = *x* in blue and linear regression through origin in red.

**Sensitivity analysis**

In order to test how sensitive the model was to changes in parameter values, the elasticity (*E*) of the total number of bird-days was calculated by varying parameters one by one. Patch dependent parameters such as attack rate or accessibility were increased or decreased for all patches simultaneously. In order to obtain one value for each parameter, year-specific parameters were averaged over the four available years, and subsequently individual parameters were decreased and increased by 20%. Elasticity was calculated using the following equation:

$E=\frac{\left( \mathrm{SD}_{\min}-\mathrm{SD}_{\max} \right)/{\mathrm{SD}_{\mathrm{ref}}}}{\left( P_{\min}-P_{\max} \right)/{P_{\mathrm{ref}}}}$where:
*D* is the number of bird-days;
*P* is a given parameter value;

and subscripts *ref* indicate the reference value, and *min* and *max* the reference value after a -20% or +20% change, respectively.

An elasticity of one means that a proportional change in parameter value changes the output with an equal proportion. A positive elasticity indicates that the output varied in the same direction as the parameter, whereas a negative elasticity indicates the opposite.

The sensitivity analysis shows that the majority of the parameters had an elasticity between 1 and -1 (Fig. S3), which means that a 20% change in parameter value caused the output to change by less than 20%. This indicates that possible uncertainties in parameter values do not have a disproportionately large impact on the output. Water depth did however have a relatively large elasticity (-2.4); when water depth rose, tuber availability was reduced. Consequently, the swans left the area earlier because the expected fitness of emigration remained unaltered. The same holds for the intake rate, that had an elasticity of 1.6; when intake rate was reduced, the swans left the system earlier because the expected fitness of emigration exceeded the fitness of the patches in the model sooner. Here it is important to note that intake rate, like daily gain, are variables comprised of several parameters, which have to change in concert to result in a proportional change of 20%. The proportion of time spent foraging (66% day^-1^), had an elasticity of -1.2; when this fraction increased, the swans reached their target energy store more quickly, and thus left the area sooner.

In general, we conclude that the model was robust to most changes, but not to changes in water depth.

**
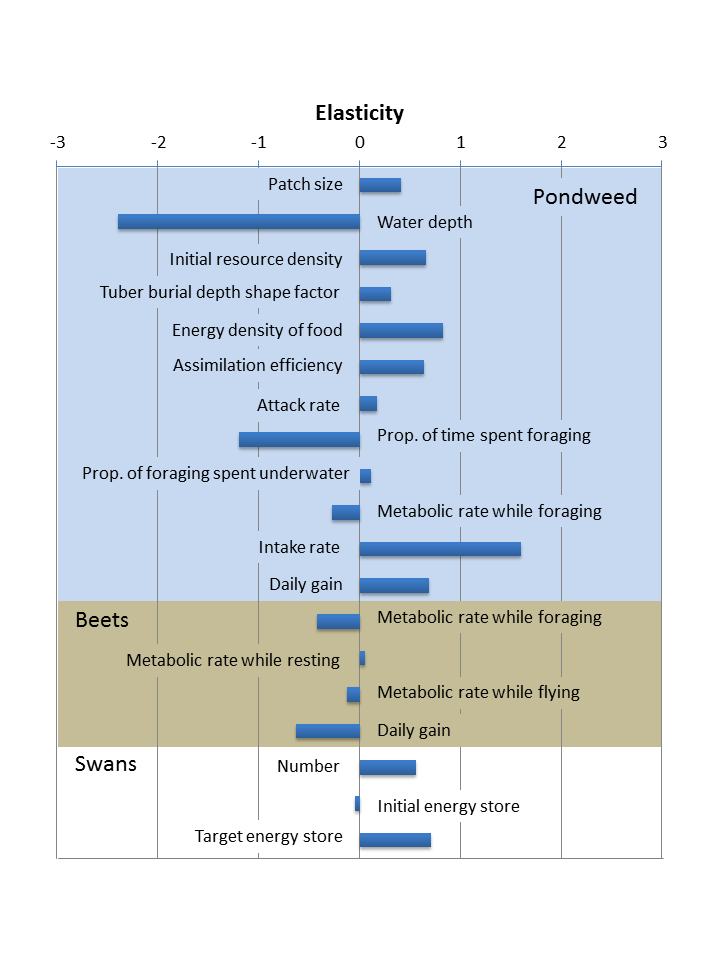
S1 Fig. C Elasticity of 19 parameters for the total number of bird-days.** The parameters are grouped in three categories, related to pondweed, beet fields and swans. Intake rate and daily gain are variables comprised of several parameters.
